# Proteome-wide dataset generated by iTRAQ-3DLCMS/MS technique for studying the role of FerB protein in oxidative stress in *Paracoccus denitrificans*

**DOI:** 10.1016/j.dib.2015.06.015

**Published:** 2015-07-06

**Authors:** Vendula Pernikářová, Vojtěch Sedláček, David Potěšil, Iva Procházková, Zbyněk Zdráhal, Pavel Bouchal, Igor Kučera

**Affiliations:** a Department of Biochemistry, Faculty of Science, Masaryk University, Kotlářská 2, 61137 Brno, Czech Republic; b Central European Institute for Technology, Masaryk University, Kamenice 5, 62500 Brno, Czech Republic; c National Centre for Biomolecular Research, Faculty of Science, Masaryk University, Kamenice 5, 62500 Brno, Czech Republic; d Regional Centre for Applied Molecular Oncology, Masaryk Memorial Cancer Institute, Žlutý kopec 7, 65653 Brno, Czech Republic

## Abstract

3DLC protein- and peptide-fractionation technique combined with iTRAQ-peptide labeling and Orbitrap mass spectrometry was employed to quantitate *Paracoccus dentirificans* total proteome with maximal coverage. This resulted in identification of 24,948 peptides representing 2627 proteins (FDR<0.01) in *P. dentirificans* wild type and *ferB* mutant strains grown in the presence or absence of methyl viologen as an oxidative stressor. The data were generated for assessment of FerB protein role in oxidative stress as published by Pernikářová et al.; proteomic responses to a methyl viologen-induced oxidative stress in the wild type and FerB mutant strains of *P. denitrificans*, J. Proteomics 2015;125:68–75. Dataset is supplied in the article.

## Specifications table

**Table:** 

Subject area	Biochemistry
More specific subject area	Proteomics
Type of data	Set of tables
How data was acquired	Liquid chromatography–tandem mass spectrometry on Orbitrap ELITE LC–MS system (Thermo Fisher Scientific)
Data format	Analyzed
Experimental factors	Bacterial cultures of *Paracocus denitrificans*, wild type and mutant in ferB gene, extracted proteins were fractionated, labeled by iTRAQ, trypsin digested, peptides were fractionated and analyzed by LC–MS
Experimental features	LC–MS analysis in HCD mode, data analysis in MaxQuant and Perseus
Data source location	Masaryk University, Brno, Czech Republic
Data accessibility	Supplementary data of the article

## Value of the data

• The highest coverage of *Paracoccus denitrificans* proteome now available [1], more than four times improved in comparison with previous analyses [2,3].
• 2627 proteins (FDR<0.01) were identified, representing 52.3% coverage of the putative proteome predicted from genome sequence.
• 34.3% coverage of membrane proteins with 1 to 25 transmembrane domains, GRAVY index of identified proteins ranged from −1.56 to 1.21.
• Physiological significance analyzed and discussed in detail by Pernikářová et al. [1].
• iTRAQ-3DLC–MS/MS technique [4] modified here is well applicable for in-depth analysis of bacterial proteomes.

## 1 Experimental design, materials and methods

### 1.1 Bacteria growth and culture conditions

Two strains of *P. denitrificans* were used in the study: Pd1222 (wt) and Pd20021 (FerB^−^). These strains were cultivated in 0.25 ml bottles filled with 45 ml of aerobic growth medium which was composed of 9 mM Na_2_HPO_4_·2H_2_O, 33 mM KH_2_PO_4_, 50 mM NH_4_Cl, 1 mM MgSO_4_·7H_2_O, 50 mM succinate and 0.033 mM ferric citrate. Rifampicin (20 µg/ml) was added to medium for wt strain growth, rifampicin (40 µg/ml) and kanamycin (25 µg/ml) were added to medium for FerB^−^ strain growth. The following culture conditions were grown: (a) wt, (b) wt with the addition of 25 µM MV (as a redox stressor), (c) FerB^−^, (d) FerB^−^ with the addition of 25 µM MV. Each of these culture conditions was cultivated in two biological replicates to gain 8 independently grown bacteria cultures. All cultures were cultivated aerobically at 30 °C from initial optical density of 0.1 till optical density of 0.6 (at 600 nm). The cells were then harvested by centrifugation for 5 min at 5000×*g*, washed once with 0.05 M NaH_2_PO_4_·2H_2_O (pH 7.3) and were stored at −80 °C.

### 1.2 Sample preparation

100 µl of lysis buffer containing 4%SDS, 50 mM NaHPO_4_ (pH 8) and 0.1 M DTT were added to each bacterial pellet. The suspension was homogenized by needle sonication (HD 2200, Bandelin, Germany, 90×0.1 s pulses at 50 W) and then heated for 5 min at 99 °C. The homogenates were then centrifuged at 16,000×*g* for 20 min at 4 °C. The supernatants (protein lysates) were precipitated overnight with 7.5 volumes of acetone at −20 °C and then centrifuged at 16,000×*g* for 20 min at 0 °C. Protein pellets were vacuum-dried in speedvac for 5 min and dissolved in 100 µl of SEC mobile phase composed of 10% methanol, 50 mM KH_2_PO_4_, 10 mM Tris 50 mM ammonium acetate, 0.3 M NaCl and 6 M guanidine hydrochloride (all chemicals from Sigma-Aldrich, St Louis, MO) for 45 min at RT, being vortexed every 15 min and then centrifuged at 16,000×*g* for 20 min at 15 °C. The protein concentration in solubilized protein extract was determined by RC-DC Protein Assay (Bio-Rad, Hercules, CA).

### 1.3 Protein fractionation by size-exclusion chromatography (SEC)

Solubilized protein extract containing 1 mg of protein (~100 µl in volume) was injected onto the SEC column (Agilent ProSEC 300S, 5 µm, 300×7.5 mm) accommodated in Agilent Infinity 1260 LC system (Agilent, Santa Clara, CA) using the flow rate 0.2 ml/min at 30 °C. The signal was monitored at 280 nm by diode array detector (DAD). The isocratic elution took 85 min and the fractions were collected in 4 chromatographic segments as follows: 1st segment was collected from 25.0 min to 38.0 min (2.6 ml in volume), 2nd segment from 38.0 min to 44.0 min (1.2 ml), 3rd segment from 44.0 min to 51.5 min (1.5 ml), 4th segment from 51.5 min to 70.0 min (3.7 ml). The protein content in the segments was determined by RC-DC Protein Assay (Bio-Rad, Hercules, CA) with two modifications: the sample volume was 50 µl and the first precipitation step was performed by adding a double volume of Reagent I. and II.

### 1.4 Trypsin digestion and iTRAQ labeling

Trypsin digestion was performed using filter aided sample preparation (FASP) protocol [5] with several modifications: The aliquots of the four segments from the SEC fractionation containing 100 µg of protein were added onto Vivacon 500 ultrafiltration spin columns (membrane cut off 10 kDa, Sartorius Stedim Biotech, Germany). The columns were centrifuged at 14,000×*g* for 45 min at 20 °C. 200 µl of 8 M urea in 0.5 M triethylammonium bicarbonate (TEAB, pH 8.5) were added onto the columns followed by centrifugation at 14,000×*g* for 15 min at 20 °C. Subsequently, 100 µl of 8 M urea in 0.5 M TEAB (pH 8.5) and 10 μl of 50 mM tris-2-carboxyethyl phosphine (TCEP) were added onto the columns. The samples were reduced for 1 h at 37 °C, the centrifugation at 14,000×*g* for 15 min at 20 °C then followed. The alkylation was performed by the addition of 5 μl of 200 mM methylmethanthiosulfonate (MMTS) with 100 µl of 8 M urea in 0.5 M TEAB (pH 8.5). The samples were then mixed in thermomixer (Eppendorf, Germany) at 600 rpm for 1 min at 25 °C, incubated for 10 min at RT without mixing and centrifuged at 14,000×*g* for 15 min at 20 °C. 100 µl of 0.5 M TEAB were added onto the columns followed by centrifugation at 14,000×*g* for 20 min at 20 °C (this step was performed twice). The digestion was performed by the addition of 3.33 µl of 1 µg/µl trypsin (AB SCIEX, Darmstadt, Germany, trypsin:protein ratio 1:30) followed by incubation for 12 h at 37 °C. The digests were collected by centrifugation at 14,000×*g* for 15 min at 20 °C and vacuum-dried to the final volume of 26 µl.

After the digestion, the iTRAQ 8-plex (AB SCIEX) labeling was performed. After adjusting pH to 7.5 by addition of 5 µl of 0.5 M TEAB, pH 8.5, four sets of iTRAQ 8-plex labels 113−121 were then added to the samples and incubated for 2 h at RT. The samples in each 8-plex were then mixed and vacuum-dried to the volume of 10 µl and stored at −80 °C.

### 1.5 Fractionation of iTRAQ labeled peptides by HILIC

The HILIC-Kinetex column (Phenomenex, Torrance, CA, 2.6 µm, 150×2.1 mm, 100 Å) accommodated in Agilent Infinity 1260 LC system was used. Mobile phase (A) was composed of 100% ACN (Merck, Germany), mobile phase (B) of water (MilliQ, Millipore) and mobile phase (C) of 50 mM ammonium formate (pH 3.2) (Sigma-Aldrich, St Louis, MO). 20 µl of mobile phase (B) were added to the sample and a sonication was performed using ultrasonic bath for 2 min. Then, 20 µl of mobile phase (A) and 5 µl mobile phase (C) were added and after further 2 min sonication the sample was centrifuged at 16,000×*g* at 20 °C for 20 min. The sample injection volume was 40 µl and the separation method was set follows: 5 min isocratic 0% B, 7 min gradient to 20% B, 23 min gradient to 34% B, 5 min gradient to 50% B, 5 min isocratic 50% B, 0.5 min gradient to 0% B and for 4.5 min isocratic 0% B; 10% mobile phase C was kept all the time. The flow rate was 0.2 ml/min, column temperature was 30 °C and the signal was monitored at 280 nm. 7–13 fractions were collected per each HILIC run (based on sample complexity). Each fraction was vacuum-dried and stored at −80 °C. Fractions collected within first 20 min of HILIC run were further cleaned by SCX chromatography to remove unreacted iTRAQ labels as described below.

### 1.6 SCX removal of unreacted iTRAQ labels

The HILIC fractions collected within first 20 min of HILIC run (see also Supplementary File 1) were reconstituted in 100 μl of mobile phase A (10 mM KH_2_PO_4_ in 25% ACN, pH 3) and sonicated using ultrasonic bath for 2 min. The SCX cartridge supplied as a part of ICAT kit (Thermo Fisher Scientific, Waltham, MA) was inserted into Agilent Infinity 1260 LC system (Agilent, Santa Clara, CA). The separation method was set as follows (flow rate 1 ml/min if not otherwise specified): 3 min 0% B (composed of 1 M KCl in mobile phase A, pH 3) at 0.5 ml/min (loading), 2 min isocratic 0% B, 2 min isocratic 35% B (elution), 2 min isocratic 100% B (cleaning), 2 min isocratic 0% B, 2 min isocratic 100% B (cleaning), 3 min isocratic 0% B (equilibration). The eluent collected during the elution step only (time 6.6–8.1 min) was vacuum-dried, reconstituted in 200 μl of 0.1% formic acid (FA), desalted on C-18 column (MicroSpin, Harvard Apparatus, Holliston, MA) as previously described, vacuum-dried and stored at −80 °C.

### 1.7 LC–MS/MS analysis

All LC–MS/MS analyses were performed by nanoscale reversed phase liquid chromatography (RSLCnano, Thermo Fisher Scientific, Waltham, MA) coupled on-line to Orbitrap Elite mass spectrometer (Thermo Fisher Scientific). Individual HILIC fractions were re-dissolved in 0.1% FA (aq) and loaded onto trap column (100 μm×30 mm) filled with sorbent X-Bridge BEH 130C18 (3.5 μm particles, Waters, Milford, MA). The peptides were eluted at 300 nl/min onto Acclaim Pepmap100 C18 analytical column (2 µm, 75 μm×250 mm; Thermo Fisher Scientific). Mobile phase A was composed of 0.1% FA and phase B was composed of ACN:methanol:2,2,2-trifluoroethanol (6:3:1; v/v/v) containing 0.1% FA. Gradient conditions were: 1% mobile phase B (0–1 min), 1–11% B (1–30 min), 11–27% B (30–60 min), 27–50% B (60–90 min), 50–95% B (90–95 min) and 95% B held for 15 min. Equilibration of trap and analytical column was performed before loading of sample into sample loop. Analytical column was coupled on-line to Nanospray Flex Ion Source (Thermo Fisher Scientific).

MS data were collected by data-dependent strategy selecting 15 precursors from MS scan (*m*/*z* range 350–2000 *m*/*z*, resolution 60,000 at *m*/*z* 400, accumulation 1×10^6^ ions for max. 200 ms, 1 microscan). 50,000 ions for max. 200 ms with isolation window of 1.3 *m*/*z* were accumulated for MS/MS spectra acquisition in Orbitrap. “Higher energy collisional dissociation” (HCD); 40% relative collision energy was used for gaining of precursor fragments and iTRAQ reporter ions. MS/MS spectra were measured with resolution 15,000 at *m*/*z* 400. Dynamic precursor exclusion was allowed for 45 s after each MS/MS spectrum measurement.

Two or three LC–MS/MS analyses were done for selected samples with sufficient sample amount and relatively high complexity. The second and the third analysis was performed with exclusion of *m*/*z* masses already assigned to peptide from target database in the previous LC–MS/MS analyses of the same sample. Mass tolerance for *m*/*z* exclusion was set to 10 ppm and retention time window to 3 min. Exclusion lists for the repeated analyses were generated using Proteome Discoverer (version 1.3, Thermo Fisher Scientific) – see Supplementary file 2 for details.

### 1.8 Proteomics data analysis

Protein identification and quantification in the iTRAQ experiment was performed with MaxQuant 1.3.0.5. (www.maxquant.org) using Andromeda database search algorithm. The data analysis parameters were: *Spectrum properties filter:* Peptide mass range: 800–7000 Da. *Peak filters:* S/N=3. *Input data: P. denitrificans* protein database downloaded from http://www.uniprot.org (2013/03/15) with 5019 protein sequences (complemented by database of common protein contaminants according to the standard Andromeda settings), enzyme name: Trypsin (cleaving polypeptides at the carboxyl side of lysine or arginine except when either is followed by proline), max. missed cleavage sites 2, taxonomy: *P. denitrificans*, strain Pd1222. *Decoy database search*: True. Peptide FDR 0.01. Protein FDR 0.01. *Tolerances*: 20 ppm/6 ppm (first search/main search) precursor mass tolerance and 20 mDa fragment mass tolerance. *Modifications:* Dynamic (variable): oxidation (M), succinylation (protein N-term). Static (fixed): iTRAQ 8-plex (K, N-term), methylthio (C).

### 1.9 Statistical analysis of proteomics data

The statistical analysis of the proteomic data was performed with Perseus 1.3.0.4. (www.maxquant.org). Proteins identified by search against decoy database, commonly occurring contaminants and proteins identified only by a modification site were removed prior to statistical analysis. The data were log 2-transformed, missing values were replaced by normal distribution and inverse logarithm of the log 2-transformed fold changes was calculated. The resulting fold changes (FCH) were considered as significant if higher than 1.50 (up-regulation) or lower than 0.67 (down-regulation). Moreover, data were statistically analyzed via two-sample t-test when effect of MV or *ferB* gene mutation was evaluated; protein level changes with *p*<0.05 were considered as statistically significant. Statistical analysis was not possible in the case of evaluation of proteins induced by MV specifically in FerB^−^ or in wt strain because of low number of observations.

## Footnotes

Appendix A Supplementary data associated with this article can be found in the online version at doi:10.1016/j.dib.2015.06.015.
